# Genome Sequence Analysis of the Oleaginous Yeast, *Rhodotorula diobovata*, and Comparison of the Carotenogenic and Oleaginous Pathway Genes and Gene Products with Other Oleaginous Yeasts

**DOI:** 10.3390/jof7040320

**Published:** 2021-04-20

**Authors:** Irene Fakankun, Brian Fristensky, David B. Levin

**Affiliations:** 1 Department of Biosystems Engineering, University of Manitoba, Winnipeg, MB R3T 2N2, Canada; fakankui@myumanitoba.ca; 2 Department of Plant Science, University of Manitoba, Winnipeg, MB R3T 2N2, Canada; brian.fristensky@umanitoba.ca

**Keywords:** lipid biosynthesis, carotenoid biosynthesis, *Rhodotorula diobovata*, oleaginous yeasts, comparative genomics

## Abstract

*Rhodotorula diobovata* is an oleaginous and carotenogenic yeast, useful for diverse biotechnological applications. To understand the molecular basis of its potential applications, the genome was sequenced using the Illumina MiSeq and Ion Torrent platforms, assembled by AbySS, and annotated using the JGI annotation pipeline. The genome size, 21.1 MB, was similar to that of the biotechnological “workhorse”, *R. toruloides*. Comparative analyses of the *R. diobovata* genome sequence with those of other *Rhodotorula* species, *Yarrowia lipolytica*, *Phaffia rhodozyma*, *Lipomyces starkeyi*, and *Sporidiobolus salmonicolor*, were conducted, with emphasis on the carotenoid and neutral lipid biosynthesis pathways. Amino acid sequence alignments of key enzymes in the lipid biosynthesis pathway revealed why the activity of malic enzyme and ATP-citrate lyase may be ambiguous in *Y. lipolytica* and *L. starkeyi*. Phylogenetic analysis showed a close relationship between *R. diobovata* and *R. graminis* WP1. Dot-plot analysis of the coding sequences of the genes crtYB and ME1 corroborated sequence homologies between sequences from *R. diobovata* and *R. graminis*. There was, however, nonsequential alignment between crtYB CDS sequences from *R. diobovata* and those from *X. dendrorhous*. This research presents the first genome analysis of *R. diobovata* with a focus on its biotechnological potential as a lipid and carotenoid producer.

## 1. Introduction

Oleaginous yeasts store more than 20% of their dry cell weight as triglycerides (TAGs). With a projected world population of 9.9 billion in 2050 [1], the course to cleaner and sustainable energy would require continuous research, development, and implementation of renewable energy strategies. The production of biofuels from oleaginous microorganisms has been widely researched as an alternative to traditional diesel from fossil fuels [2,3]. In addition to their application in biodiesel production, some oleaginous microorganisms have other biotechnological applications in nutritional and nutraceutical supplements, cosmetics, and medical and lipid research [4]. Some oleaginous yeasts are amenable to genetic engineering manipulations. In fact, *Rhodotorula toruloides* has been dubbed a “workhorse” for biotechnological applications [5]. While *R. toruloides* has been widely studied and engineered for various applications, other *Rhodotorula* species remain under characterized. One of those is the carotenogenic and oleaginous yeast, *Rhodotorula diobovata*.

*Rhodotorula diobovata* (Basionym, *Rhodosporidium diobovatum*) is an oleaginous, marine red yeast that can concomitantly synthesize quality single-cell oils for biodiesel production and health-promoting compounds, carotenoids, which are useful as food colorants in the food industry [6]. *R. diobovata* is capable of producing high biomass, with high cell-specific yields of triacylglycerides (TAGs) when grown on waste substrates such as biodiesel-derived waste glycerol [7]. This character makes this yeast even more attractive, because a reduction in the cost of the substrate will have a positive effect on the cost of biodiesel production. *R. diobovata* has been used in bioremediation, as it is able to assimilate high concentrations of nitrogen [8], and it has been found to thrive under varying culture conditions [9,10]. This study presents the first whole-genome sequence analysis and annotation of *R. diobovata* and compares the genome of *R. diobovata* with other oleaginous and carotenogenic yeasts with a focus on their lipid and carotenoid biosynthesis pathways. 

## 2. Materials and Methods

### 2.1. Microorganism and DNA Extraction

*Rhodotorula diobovata,* UCDFST 08-225, was obtained from the Phaff Yeast Culture Collection, University of California, Davis (UCDFST). Cells were revived in YPD broth (20 g/L dextrose, 10 g/L yeast extract, and 20 g/L peptone) and incubated at 30 °C for 48 h (h). The inoculum was streaked on YPD agar plates containing 20 g/L dextrose, 10 g/L yeast extract, 20 g/L peptone, and 15 g/L agar and incubated at 30 °C for 24 h. Colonies from the agar plates were directly inoculated into YPD broth containing 10 g/L yeast extract, 20 g/L peptones, and 20 g/L glucose and grown at 30 °C and 150 rpm for 24 h. 

To extract genomic DNA, 10 mL of culture was transferred into a falcon tube and centrifuged at 3000× *g* for 5 min at 4 °C. Cell pellets were resuspended in phosphate buffer (PBS) and washed by centrifugation, followed by resuspension in PBS three times. A small aliquot (500 µL) of cells was transferred to screw-capped Eppendorf for cell rupture. Cell rupture was carried out by bead-beating with about 20 mg of 0.25 mm glass beads in a rotary bead-beater (Fisherbrand Bead Mill 24 Homogenizer) with three to five 30 s (s) cycles. To separate the debris from the lysate, tubes were centrifuged at 16,000× *g* for 20 min at 4 °C. DNA extraction and purification were conducted using the Promega Wizard^®^ Genomic DNA Purification Kit (Promega, Madison, WI, USA). DNA extraction was done in triplicate. Concentrations of the resulting sample were measured using Thermo Scientific’s NanoDrop 1000 spectrophotometer (Thermo Fisher Scientific Inc., Madison, WI, USA).

### 2.2. Genome Sequencing and Annotation

The genome of the haploid strain, *R. diobovata* 08-225, was sequenced using the Ion Torrent (IT) PGM and Illumina Miseq platforms. A total of four libraries were constructed, resulting in 3,051,898 reads from the IT platform, as well as 9,949,684 reads, 8,240,002 reads, and 5,899,760 reads from three Illumina libraries. The Illumina libraries had insert sizes of 300, 400, and 700 bp, while the IT library was 500 bp. The quality of the sequences was assessed using FastQC, and read error correction was carried out by Quake and Pollux error correction software. The Abyss Genome assembler resulted in the best hybrid assembly of Quake-corrected sequences from the Illumina platform and Pollux-corrected sequences from the IT platform [11,12]. 

To obtain a more reliably annotated genome, with information on exons, splicing sites, and alternative splicing, RNA-Seq was carried out on samples from mid-log and early stationary phase cells using the Illumina HiSeq platform. Transcripts were de novo assembled using rnaSPAdes and Trinity combined with two read-trimming tools: Trim Galore and Trimmomatic. Transrate was used to evaluate the quality of the assemblies. The rnaSPAdes assembler produced the best-quality assembly with samples from the mid-log phase giving a transrate score of 0.08. 

The assembled genome and transcriptome were submitted to the Joint Genome Institute’s (JGI) MycoCosm platform and annotated using their annotation pipeline [13]. The annotated genome was further subjected to manual curation using Artemis [14]. This Whole-Genome Shotgun project has been deposited at DDBJ/ENA/GenBank under the accession SOZI00000000. The version described in this paper is version SOZI01000000.

### 2.3. Comparative Genomics and Phylogenetic Analyses

Sequence datasets were created for proteins of interest, including malic enzyme, ATP-citrate lyase, acetyl-CoA acetyltransferase, bifunctional lycopene cyclase/phytoene synthase, and phytoene desaturase by BLASTP search. Phylogenetic analyses between these metabolic enzymes were used to infer sequence similarity among other oleaginous yeasts. Differences in the activity of these enzymes reported in the literature for some oleaginous yeasts [15,16] informed the choice of oleaginous yeast sequences included in the phylogenetic analysis. Prior to multiple sequence alignments, redundant sequences were eliminated from the dataset using cd-hit (http://cd-hit.org). The sequences were then aligned by the MAFFT multiple sequence alignment program using the iterative refinement method FFT-NS-i [17]. Phylogenetic trees were constructed using protein maximum likelihood (PROML), and statistical support was obtained by a bootstrapping method 100 times. LAST was used to align DNA sequences in select scaffolds in *R. graminis* versus *R. diobovata*. All programs were accessed using the comprehensive bioinformatics system, BIRCH [18]. 

## 3. Results and Discussion

### 3.1. General Features of the Rhodotorula Diobovata Genome

Statistics of the genome assembly are shown in Table 1 (scaffold length distributions and genome coverage calculations are shown in Tables S1 and S2). De novo assembly of *R. diobovata* 08-225 produced 361 scaffolds, with the largest scaffold being 0.56 Mbp. The scaffold L50 number was 0.12 Mbp, the GC content was 66.9%, and the size of the genome was 21.1 MB, which is similar to the genome size of other red oleaginous yeasts. *R. graminis* WP1 has a genome size of 21.01 MB [19], *R. glutinis* ATCC 204,091 has a size of 20.48 MB, *R. toruloides* NP11 is 20.22 MB [20], and *Rhodotorula* sp. JG-1b has a genome size of 19.39 MB [21]. The draft genome sequence of *R. diobovata* annotated by the JGI pipeline is estimated to have a total of 7970 protein-coding genes with an average of 5.95 exons per gene and an average protein length of 510 amino acids. While yeasts such as *Saccharomyces cerevisiae* generally have about 5800 coding genes with an average of 1 exon per gene, it is usual for *Rhodotorula* species to have a higher number of protein-coding genes and exons per gene. For example, 8171 protein-coding genes were predicted in *R. toruloides* NP11, with an average of 6.26 exons per gene [13].

### 3.2. Analysis of Proteins Associated with De Novo Fatty Acid Biosynthesis

The biosynthesis of fatty acids in oleaginous yeasts may be either de novo or ex novo [22,23]. This study focuses on the de novo biosynthesis pathway, which involves the fermentation of sugars and related substrates. Biochemically, the “oleaginicity” of a microorganism is defined by the presence of certain key enzymes: isocitrate dehydrogenase, adenosine triphosphate (ATP)-citrate lyase, malic enzyme, and fatty acid synthase [24]. ATP-citrate lyase is responsible for providing acetyl subunits for fatty acid biosynthesis. Malic enzyme is involved in the generation of nicotinamide adenine dinucleotide phosphate (NADPH) to reduce acetyl subunits, which form the backbone of fatty acids [25]. Here, ATP-citrate lyase and malic enzyme from *R. diobovata* are analyzed, and datasets for sequence alignment were created to include sequences from other oleaginous yeasts. 

### 3.3. Malic Enzyme

Malic enzyme (ME) catalyzes the oxidative decarboxylation of malate to pyruvate, producing NADPH, which is necessary for fatty acid biosynthesis. Ratledge [26] reviewed the role of ME as a producer of NADPH in oleaginous microorganisms and concluded that ME could not be the only source of NADPH in oleaginous microorganisms, because some oleaginous microorganisms, such as *Y. lipolytica* and *L. starkeyi*, were found to possess just one malic enzyme present in the mitochondria [27]. Fatty acid biosynthesis is a cytosolic process, and the absence of a cytosolic malic enzyme in these yeasts suggests that the supply of NADPH for fatty acid biosynthesis may be from alternative pathways, such as the pentose phosphate pathway. 

**Figure 1:**
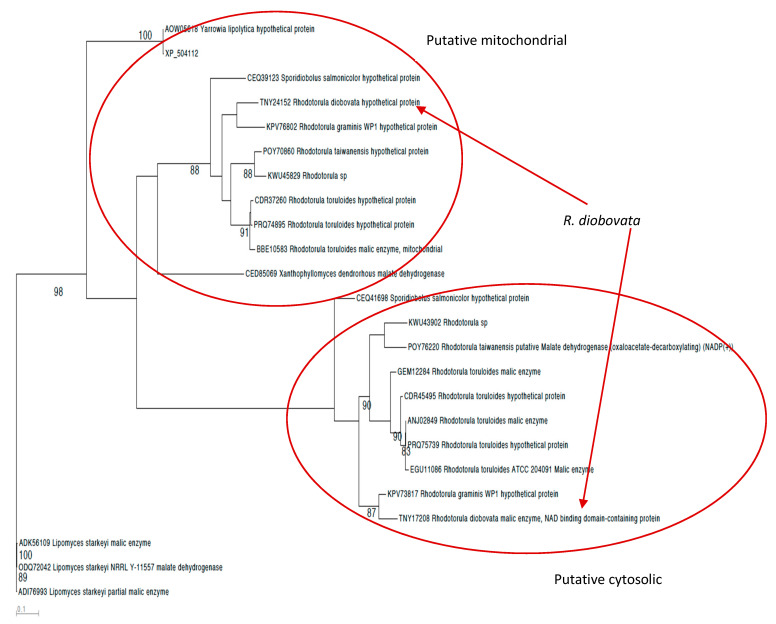
PROML phylogenetic tree constructed from an alignment of malic enzyme sequences from *Rhodotorula* species, *Yarrowia, Lipomyces*, and *Xanthophyllomyces. Rhodotorula diobovata* sequences are pointed out, showing cytosolic and mitochondrial positioning. The significant nodes on the tree with bootstrap support values >70% are highlighted.

*R. diobovata*, like many other *Rhodotorula* species, has 2 MEs (DMC30DRAFT_420609 and DMC30DRAFT_357466) with predicted lengths of 633 and 613 amino acids, respectively. This is similar to the number of proteins annotated as ME in the oleaginous yeast *Papiliotrema laurentii* [16] but dissimilar to the ten genes annotated as encoding ME in *Trichosporon fermentans* [28]. Phylogenetic analyses based on the MAFFT alignment of amino acid sequences of malic enzyme revealed that MEs from *R. diobovata* (TNY17208 and TNY24152) were most closely related with those from *R. graminis WP1* (KPV73817 and KPV76802) (Figure 1).

**Figure 2:**
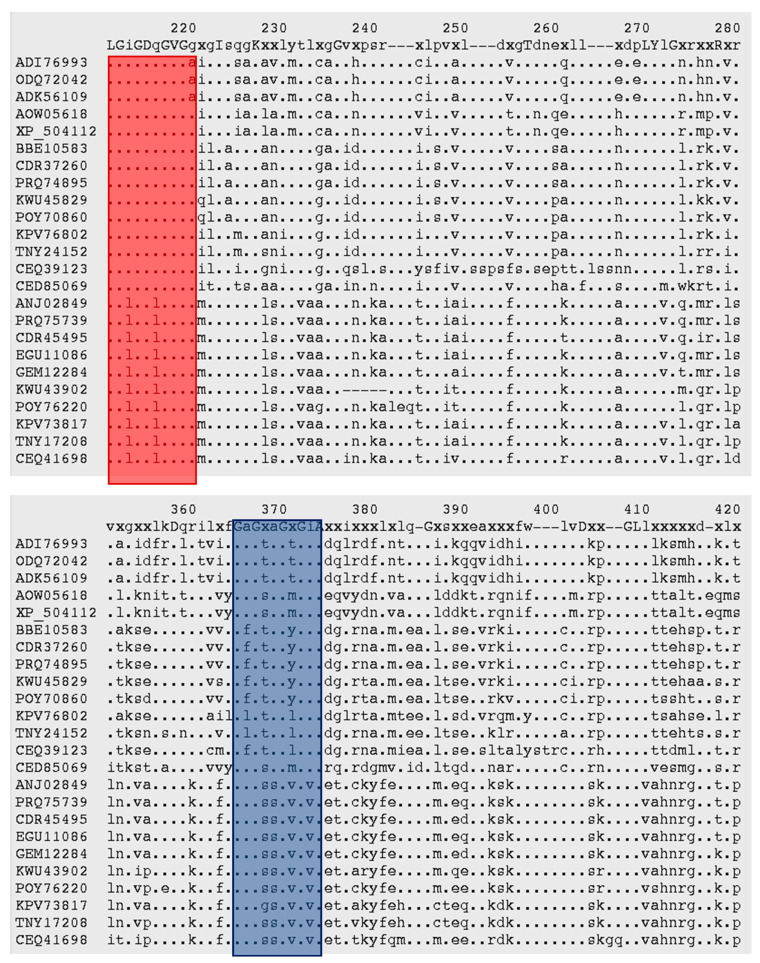
MAFFT alignment of malic enzymes (MEs) showing dinucleotide-binding sites. The red box is for NAD-linked MEs, and the blue box is for NADP-linked MEs [29]. Identical amino acid residues in all the enzymes are signified by a dot (.) ADI76993, *Lipomyces starkeyi*; ODQ72042, *Lipomyces starkeyi* NRRL Y-11557; ADK56109, *Lipomyces starkeyi*; AOW05618, *Yarrowia lipolytica* CLIB89(W29); XP_504112, *Yarrowia lipolytica* CLIB122; BBE10583, *Rhodotorula toruloides* NBRC10032; CDR37260, *Rhodotorula toruloides* CECT1137; PRQ74895, *Rhodotorula toruloides* NBRC0880; KWU45829, *Rhodotorula* sp. JG-1b; POY70860, *Rhodotorula taiwanensis*; KPV76802, *Rhodotorula graminis* WP1; TNY24152, *Rhodotorula diobovata*; CEQ39123, *Sporidiobolus salmonicolor*; CED85069, *Xanthophyllomyces dendrorhous*; ANJ02849, *Rhodotorula toruloides* IFO0880; PRQ75739, *Rhodotorula toruloides* NBRC0880; CDR45495, *Rhodotorula toruloides* CECT1137; EGU11086, *Rhodotorula toruloides* ATCC 204091; GEM12284, *Rhodotorula toruloides* NBRC10032; KWU43902, *Rhodotorula* sp. JG-1b; POY76220, *Rhodotorula taiwanensis*; KPV73817, *Rhodotorula graminis* WP1; TNY17208, *Rhodotorula diobovata*; CEQ41698, *Sporidiobolus salmonicolor*.

A WebLogo plot of the aligned sequences (Figure S1) was constructed to identify the regions where specific amino acids were conserved before a more in-depth analysis of the alignment was carried out. In addition to the divalent ion-binding sites (Figure S2), alignment analysis revealed two dinucleotide-binding sites associated with NAD and NADP. These conserved regions (highlighted in Figure 2) have glycine-rich sequences [29,30]. Sequence similarities within the putative mitochondrial putative cytosolic enzymes are obvious in the NAD-linked and NADP-linked regions. The putative cytosolic enzymes have aliphatic and nonpolar leucine (L) at Position 216 instead of the acidic and polar glutamine (Q) observed in the putative mitochondrial enzymes at the NAD-linked region. Position 369 of the alignment shows all of the putative cytosolic enzymes contained hydroxylic and polar serine (S) instead of aliphatic and nonpolar alanine (A) observed in the mitochodrial enzymes. For the NADP-linked region, *Y. lipolytica*, *L. starkeyi*, and *X. dendrorhous* have a hydroxyl or sulfur-containing amino acid at Position 371 of the alignment rather than the aliphatic or aromatic amino acids (valine, leucine, and tyrosine) observed in the same position for puciniomycotas examined in this analysis. 

While *X. dendrorhous* is not established as an oleaginous yeast, *Y. lipolytica* and *L. starkeyi* are established oleaginous yeasts, the ME activities of which have been unclear, as they do not possess cytosolic ME. In fact, the sequence in the NADP-ME-conserved regions has been associated with *Y. lipolytica*’s preference of NAD^+^ over NADP^+^ as a cofactor [15]. Zhang et al. [15] found that NADP^+^-dependent activity was only 1% of NAD^+^-dependent activity and that NAD^+^ had a much lower Km value. Another alternative for NADPH supply in *Y. lipolytica* and *L. starkeyi* is the cytosolic NADP^+^-dependent isocitrate dehydrogenase [26].

### 3.4. ATP-Citrate Lyase

ATP-citrate lyase (ACL), also annotated as ATP-citrate synthase, is an enzyme characteristic of oleaginous microorganisms. While the presence of ACL does not confer oleaginicity, oleaginous microorganisms have been found to possess it [31]. ACL cleaves citrate in the cytosol to produce acetyl-CoA and oxaloacetate. The alignment of *R. diobovata* ACL amino acid sequences with those from other oleaginous yeasts shows a close relationship between *R. diobovata* and *R. toruloides* NBRC10032 (Figure S3). This analysis shows that *Rhodotorula* species appear to have only one gene encoding the ACL enzyme, unlike the oleaginous *Saccharomycotina* species, which possess two genes encoding two ACL subunits, while eight (8) genes encoded for ACL in *T. fermentans*, six (6) in *A. oryzae*, and seven (7) in *R. opacus* genomes [28]. 

**Figure 3:**
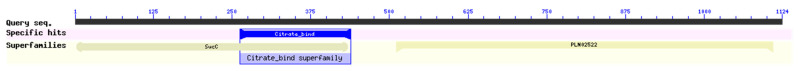
Graphical summary of conserved domains on ATP-citrate synthase in *Rhodotorula diobovata* obtained from the Conserved Domain Database [34].

The protein ATP-citrate synthase in *R. diobovata* has 1124 amino acids, with 3 conserved regions: succinyl-CoA synthetase (SucC) beta subunit (cl33750). ATP-citrate lyase citrate-binding (Citrate_bind superfamily) (cl29684), and ATP-citrate (pro-S)-lyase (PLN02522) (cl31895) (Figure 3). Other *Rhodotorula* species analyzed only contained the cl31895 and cl29684 conserved regions on one ACL (Figure 3). SucC is, however, a conserved domain containing the citrate-binding sites cl29684 and cl33750, which are, therefore, overlapping homologous superfamilies [32]. Oleaginous *Saccharomycotina*, which includes *Y. lipolytica* and *L. starkeyi*, have conserved domains cl29684 and cl31895 on separate subunits. This may explain the difference in ACL expressions under lipogenic conditions. While ACL gene expressions did not correlate with the lipid accumulation phase in *Y. lipolytica* [33], expression levels of ACL in *R. toruloides* were high in the lipid accumulation phase [20].

### 3.5. Analysis of Proteins Associated with Carotenoid Biosynthesis

**Figure 4:**
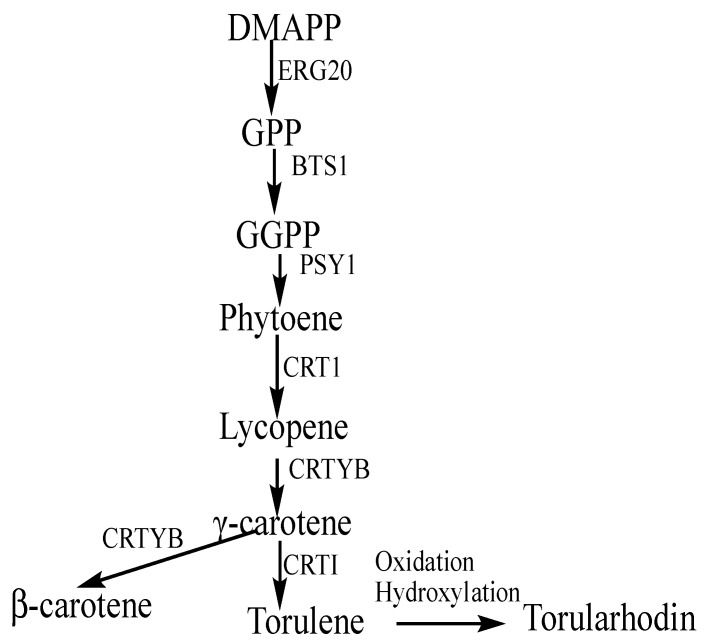
Isoprenoid biosynthesis pathways. DMAPP, dimethylallyl pyrophosphate; ERG20, farnesyl pyrophosphate synthetase; GPP, geranyl pyrophosphate; BTS1(CRTE), geranylgeranyl pyrophosphate synthase; GGPP, geranylgeranyl pyrophosphate; PSY1(CRTB), phytoene synthase; CRTI, phytoene desaturase; CRTYB (CRTY), bifunctional lycopene cyclase/phytoene synthase.

Carotenoid biosynthesis is divided into three steps: the synthesis of mevalonate, followed by the synthesis of isopentenyl pyrophosphate (IPP), which leads to the biosynthesis of terpenoids [35]. The first step in the biosynthesis of mevalonate in red oleaginous yeasts is the formation of acetoacetyl-CoA from acetyl-CoA in the cytosol. This is achieved by the action of a thiolase, acetyl-CoA C-acetyltransferase (ACAT), which catalyzes the transfer of an acetyl group to acetyl-CoA. The addition of another acetyl group catalyzed by 3-hydroxy-3-methylglutaryl-CoA (HMG-CoA) synthase is the second step in mevalonate biosynthesis. Another acetyl group is then added to 3-hydroxy-3-methylglutaryl-CoA synthesized in the second step. This reaction is catalyzed by HMG-CoA reductase, and it is considered the rate-limiting step in carotenoid biosynthesis [36]. IPP is synthesized by a series of phosphorylation reactions that lead to the final stage of terpenoid biosynthesis (Figure 4). 

*Rhodotorula* species typically synthesize β-carotene, torulene, and torularhodin [37,38]. However, *R. diobovata* favors the biosynthesis of torulene and torularhodin over β-carotene under varying growth conditions [6]. In addition to the comparative analysis of thiolase, the first enzyme in the biosynthetic pathway of carotenoids, an assessment of bifunctional lycopene cyclase/phytoene synthase and phytoene desaturase was carried out. 

### 3.6. Acetyl-CoA C-Acetyltransferase

Acetyl-CoA C-acetyltransferase (encoded by ERG10) belongs to the ubiquitous thiolase family, and it catalyzes the reaction from acetyl-CoA to acetoacetyl-CoA. There are two broad types of thiolase-degradative thiolases (3-ketoacyl-CoA thiolase) and biosynthetic thiolases (acetoacetyl-CoA thiolase), which are involved in the first step of mevalonate synthesis [39]. While there are five (5) distinct genes encoding thiolase enzymes in the *R. diobovata* (Accession Numbers: TNY24221, TNY23669, TNY19355, TNY19167, TNY16984), TNY19355 revealed the best alignment with the thiolase-conserved domain, cd00751, with an E-value of 0. 

Alignment analysis of acetyl-CoA C-acetyltransferase from *R. diobovata, P. rhodozyma, R. toruloides*, *S. salmonicolor*, *R. graminis*, and *R. taiwanensis* shows the two conserved cysteine residues at the N-terminus and C-terminus involved in the acyl-enzyme intermediate formation and active site base, respectively [40]. Phylogenetic analyses revealed a close relationship between TNY19355 (*R. diobovata*) and KPV74338 (*R. graminis* WP1) (Figure S4).

### 3.7. Bifunctional Lycopene Cyclase/Phytoene Synthase

**Figure 5:**
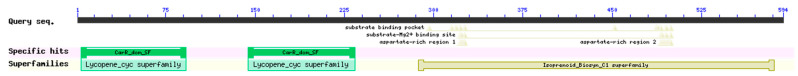
Graphical summary of putative conserved domains of the bifunctional lycopene cyclase/phytoene synthase of *Rhodotorula diobovata* obtained from the Conserved Domain Database [34].

Bifunctional lycopene cyclase/phytoene synthase is responsible for phytoene synthesis, the cyclization of lycopene to γ-carotene, and further cyclization to β-carotene (Figure 4). The gene coding for this bifunctional enzyme in *R. diobovata* crtYB was studied by Guo et al. [41]. The protein contains two copies of the CarR_dom_SF domain, which is the lycopene cyclase domain, on the N-terminus. The Isoprenoid_Biosyn_C1 (phytoene synthase) superfamily domain is located at the C-terminal (Figure 5). Phylogenetic analyses of the 594 amino acid sequence of crtYB from *R. diobovata* (AGT42003) with those from other *Rhodotorula* species, *P. rhodozyma, S. salmonicolor*, and *S. pararoseus*, revealed that the *R. diobovata* crtYB was most closely related to the crtYB of *R. graminis* WP1. An increase in the expression of this gene has been linked to increased carotenoid production in *R. toruloides* under light conditions [42], and it has been shown to be transcriptionally regulated in *X. dendrorhous* [43]. This gene is also one of the most favored in the engineering of noncarotenogenic yeast strains for the production of β-carotene [44]. 

### 3.8. Phytoene Desaturase

Phytoene desaturation is a rate-limiting step in the carotenoid biosynthetic pathway [45]. Phytoene desaturase (phytoene dehydrogenase), encoded by the *crtI* gene, catalyzes the formation of lycopene from phytoene. The same enzyme also catalyzes the formation of torulene from γ-carotene (Figure 4). Phytoene desaturase (PDS), TNY20974 (551 aa) from *R. diobovata* UCDFST 08-225 with a conserved region, crtI_fam, spanning Positions 7 to 505, was aligned with the enzyme from other Basidiomycota species, and this alignment revealed a 99% amino acid (aa) sequence identity with a previously characterized PDS from *R. diobovatum* ATCC 2527. Guo et al. [46] found that this *crtI* gene encodes a 547 amino acid PDS protein with a molecular mass of 60.45kDa and has 7 introns. Its amino acid sequence (AHB14354) was shown to have a 46% aa sequence identity with the *X. dendrorhous* PDS enzyme and a 45% aa sequence identity with the PDS of *Blakeslea trispora*, while TNY20974 from this work showed a 47% aa sequence identity with the PDS of *X. dendrorhous.* Phylogenetic analysis showed a close relationship between the *R. diobovata* PDS amino acid sequences and the PDS from *R. graminis* WP1. 

**Figure 6:**
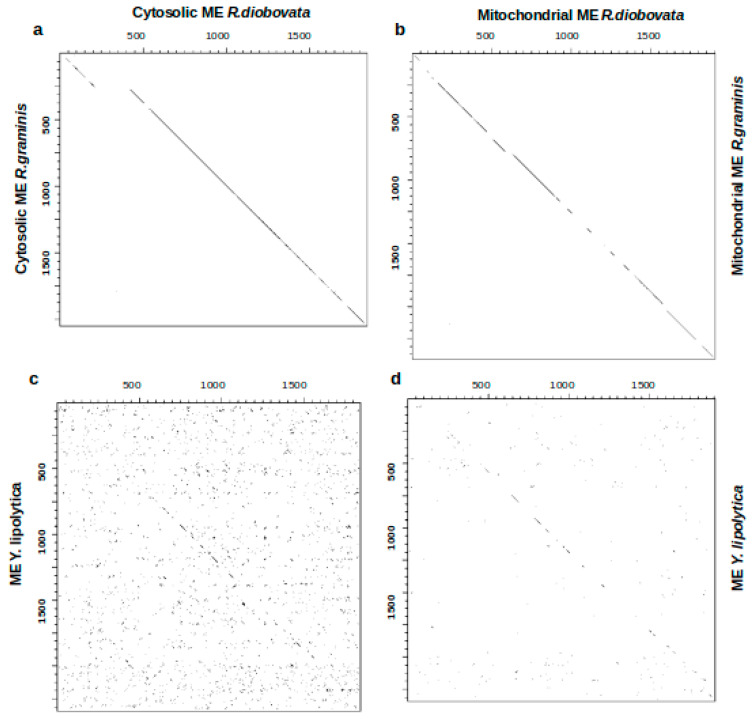
Dot-plots of individual protein-coding sequences constructed by dotter showing mostly a continuous match between sequences. (**a**) Cytosolic ME in *Rhodotorula diobovata* on the *x*-axis and cytosolic ME in *Rhodotorula* graminis on the *y*-axis; (**b**) mitochondrial ME in *R. diobovata* on the *x*-axis and mitochondrial ME in *R. graminis* on the *y*-axis; (**c**) cytosolic ME in *R. diobovata* on the *x*-axis and ME in *Yarrowia lipolytica* on the *y*-axis; (**d**) mitochondrial ME in *R. diobovata* on the *x*-axis and *Y. lipolytica* ME in on the *y*-axis.

**Figure 7:**
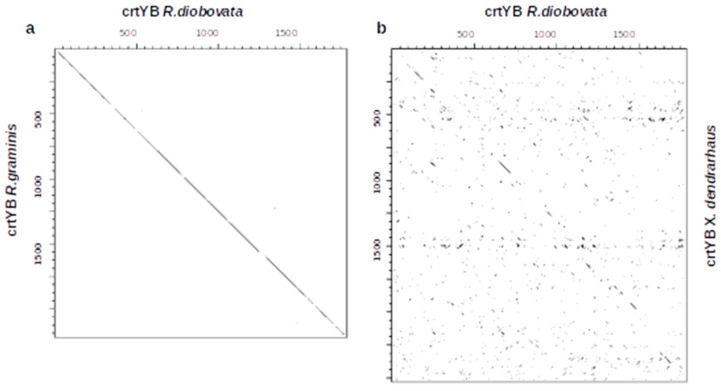
Dot-plots of individual protein-coding sequences constructed by dotter showing (**a**) a continuous sequence match of crtYB in *Rhodotorula diobovata* on the *x*-axis and crtYB sequence from *Rhodotorula graminis* on the *y*-axis and (**b**) regions that are strongly conserved between crtYB of *R. diobovata* and the crtYB sequence from *Xanthophyllomyces dendrorhous*, with little evident conservation in the rest of the lengths of the two protein-coding sequences.

From the sequence analysis of enzymes in the lipid and carotenoid biosynthesis pathways, there is a general close alignment between *R. graminis* WP1 and *R. diobovata*. To further examine the sequence similarities observed between *R. graminis* WP1 and *R. diobovata,* dot-plot analysis of the coding sequences (CDS) of crtYB and ME genes from these yeasts was constructed. CDS of ME and crtYB from *Y. lipolytica* and *X. dendrorhous*, respectively, were also included in this analysis. The dot-plot was used to visually inspect sequences, and it revealed similarities and some frameshifts (Figure 6 and Figure 7). Cytosolic and mitochondrial ME sequences of *R. diobovata* and *R. graminis* show sequence homology evident from the continuous match. There is, however, an insertion observed between the two cytosolic sequences (Figure 6a) [47]. 

Cytosolic and mitochondrial ME sequences of *R. diobovata* were also plotted against CDS of ME from *Y. lipolytica* (Figure 6c,d), which is localized in the mitochondria. The organizational difference observed here may be responsible for how malic enzyme operates in *Y. lipolytica* compared to other oleaginous yeasts. Dot-plots of CDS sequences of crtYB of *R. diobovata* versus *R. graminis* also showed a continuous match between the two sequences (Figure 7a). Sequences from *R. diobovata* versus *X. dendrorhous*, however, appear to have a series of sequence deletion events (Figure 7b). The study of these types of insertions, deletions, and rearrangements is important, as they may alter phenotypes. Despite the phenotypes of oleaginous and carotenogenic yeasts being similar, genome and sequence-structure data can provide useful information needed prior to gene editing. This then forms a basis for genetic engineering for biotechnological applications.

## 4. Conclusions

This study presented the whole-genome sequencing of an oleaginous and carotenogenic basidiomycete, *Rhodotorula diobovata*, and revealed core differences between the amino acid sequences of *R. diobovata* proteins involved in the lipid and carotenoid biosynthesis pathways and those from other oleaginous yeasts. The unconventional mode of action of malic enzyme and ATP-citrate lyase in *L. starkeyi* and *Y. lipolytica* may be connected to their sequence organization. Dot-plot analysis of the coding sequences of malic enzyme and crtYB confirmed sequence homologies between *R. diobovata* and *R. graminis* sequences. There was, however, nonsequential alignment between crtYB CDS sequences from *R. diobovata* and those from *X. dendrorhous*. This research presents useful information for genetic engineering and the potential exploration of *R. diobovata* as a biotechnological “workhorse”. 

## Figures and Tables

**Table 1:** Summary of genome assembly statistics.

Genome assembly size (Mbp)	21.14
Genome coverage	44×
Number of contigs	614
Number of scaffolds	361
Number of scaffolds ≥2Kbp	336
Scaffold N50	55
Scaffold L50 (Mbp)	0.12
Number of gaps	253
Percentage of scaffold length in gaps	0.10%
Three largest scaffolds (Mbp)	0.56, 0.49, 0.41

## Data Availability

This Whole-Genome Shotgun project has been deposited at DDBJ/ENA/GenBank under the accession SOZI00000000. https://www.ncbi.nlm.nih.gov/bioproject/?term=rhodotorula%20diobovata (accessed on 19 June 2019).

## Supplementary Materials

The following are available online at https://www.mdpi.com/article/10.3390/jof7040320/s1, Figure S1: Weblogo plot of aligned sequences from Rhodotorula diobovata, Lipomyces starkeyi, Rhodotorula toruloides NP11, Rhodotorula graminis WP1, *Rhodotorula* sp. JG-1b, Sporidiobolus salmonicolor, Phaffia rhodozyma, Yarrowia lipolytica, and Rhodotorula taiwanensis. Plot shows relative frequency of each amino acid at each position. Red highlighted boxes show the dinucleotide binding sites while the blue boxes show the divalent ion binding sites. Figure S2. MAFFT alignment of malic enzymes showing the divalent ion binding sites [Borsch and Westhoff, 1990]. Identical amino acid residues in all the enzymes are signified by a dot (.) ADI76993, *Lipomyces starkeyi; ODQ72042, Lipomyces starkeyi NRRL Y-11557; ADK56109, Lipomyces starkeyi; AOW05618, Yarrowia lipolytica CLIB89(W29); XP_504112, Yarrowia lipolytica CLIB122; BBE10583, Rhodotorula toruloides NBRC10032; CDR37260, Rhodotorula toruloides CECT1137; PRQ74895, Rhodotorula toruloides NBRC0880; KWU45829, Rhodotorula sp. JG-1b; POY70860, Rhodotorula taiwanensis; KPV76802, Rhodotorula graminis WP1; TNY24152, Rhodotorula diobovata; CEQ39123, Sporidiobolus salmonicolor; CED85069, Xanthophyllomyces dendrorhous; ANJ02849, Rhodotorula toruloides IFO0880; PRQ75739, Rhodotorula toruloides NBRC0880; CDR45495, Rhodotorula toruloides CECT1137; EGU11086, Rhodotorula toruloides ATCC 204091; GEM12284, Rhodotorula toruloides NBRC10032; KWU43902, Rhodotorula sp. JG-1b; POY76220, Rhodotorula taiwanensis; KPV73817, Rhodotorula graminis WP1; TNY17208, Rhodotorula diobovata; CEQ41698, Sporidiobolus salmonicolor.* Figure S3. PROML phylogenetic tree constructed from an alignment of ATP: Citrate lyase sequences from *Rhodotorula* sp., Yarrowia, Lipomyes, and Xanthophyllomyces. Analysis shows the relationship between the two ACL subunits of *Y. lipolytica* and *L. starkeyi*. The significant nodes on the tree with bootstrap support values > 70% are highlighted. Figure S4. PROML phylogenetic tree constructed from a MAFFT alignment of Acetyl-CoA C-acetyltransferase sequences from Rhodotorula species, Yarrowia, Lipomyes, Sporidobolus and Xanthophyllomyces. The significant nodes on the tree with bootstrap support values > 70% are highlighted. Figure S5. Scaffold length distribution of all 361 scaffolds from the De novo assembly of *R. diobovata* 08-225. Table S1. Scaffold length distribution for the 361 scaffolds used to assemble the *Rhodotorula diobovata* genome Table S2. Genome coverage calculation for Illumina and Ion Torrent reads.
