# Supplementary material for: Genome Sequence Analysis of the Oleaginous Yeast, Rhodotorula diobovata, and Comparison of the Carotenogenic and Oleaginous Pathway Genes and Gene Products with Other Oleaginous Yeasts
Source: J Fungi (Basel). 2021 Apr 20;7(4):320. doi: 10.3390/jof7040320 (PMC8074367; doi:10.3390/jof7040320)
Supplement: Supplementary file 1 [file jof-07-00320-s001.zip › jof-1173790-supplementary.pdf]

Supplementary Data

Genome sequence analysis of the oleaginous yeast, *Rhodotorula diobovata*, and comparison of the carotenogenic and oleaginous pathway genes and gene products with other oleaginous yeasts.

Irene Fakankun[1], Brian Fristensky[2], David B. Levin[1*]

[1]Department of Biosystems Engineering and [2]Department of Plant Science,
University of Manitoba, Winnipeg, Manitoba, Canada, R3T 2N2

* Corresponding Author: Tel: 204-474-7429; Fax: 204-474-7512; E-mail: david.levin@umanitoba.ca

Irene Fakankun: fakankui@mymanitoba.ca
Brian Fristensky: brian.fristensky@umanitoba.ca

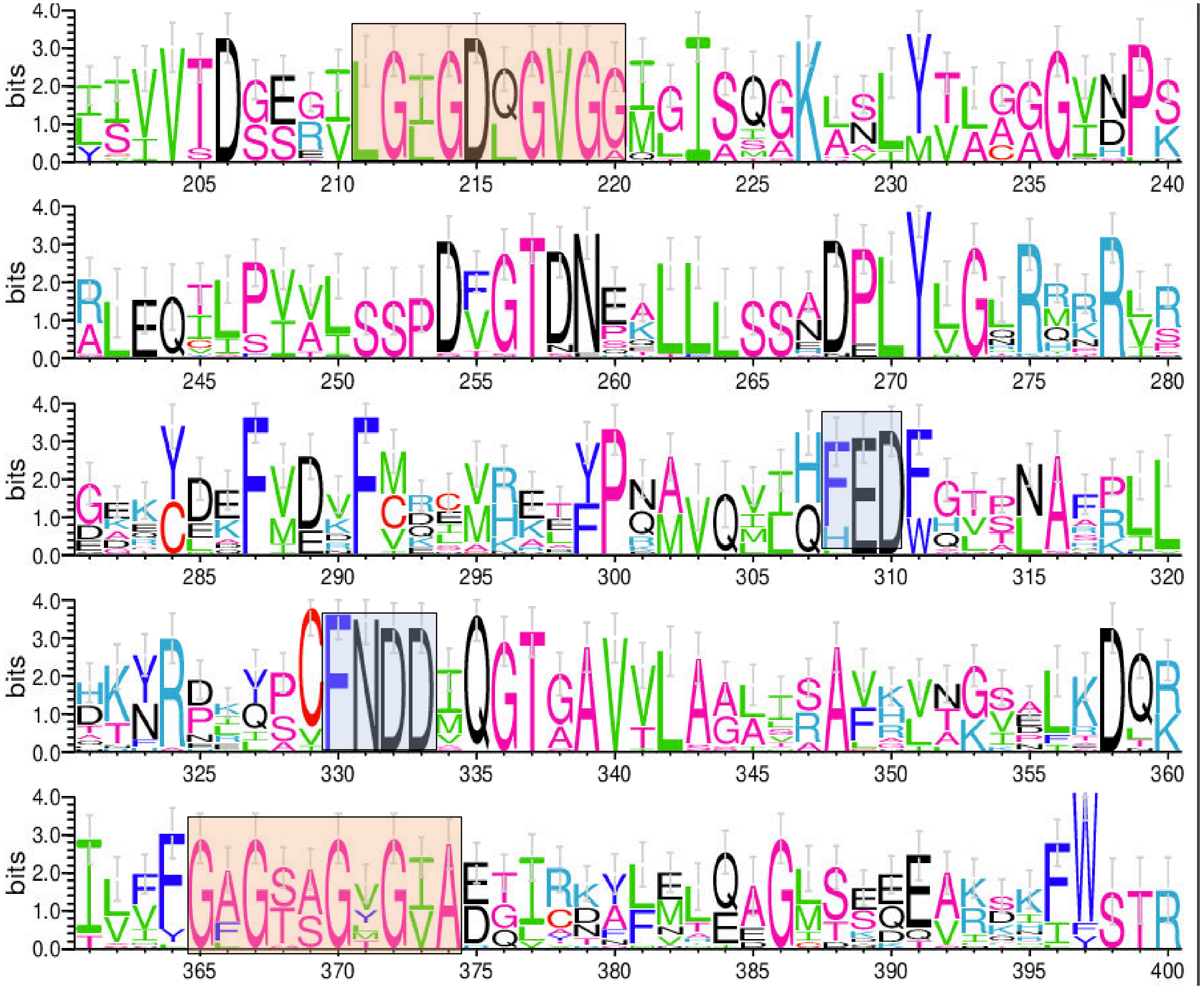

Figure S1. Weblogo plot of aligned sequences from *Rhodotorula diobovata*, *Lipomyces starkeyi, Rhodotorula toruloides NP11, Rhodotorula graminis WP1, Rhodotorula sp. JG-1b, Sporidiobolus salmonicolor, Phaffia rhodozyma, Yarrowia lipolytica, and Rhodotorula taiwanensis*. Plot shows relative frequency of each amino acid at each position. Red highlighted boxes show the dinucleotide binding sites while the blue boxes show the divalent ion binding sites.

```
                   290       300       310       320       330       340       350
           gxxydxFvdxFxxxxxxxyPxa--xihfEDfxxxnaxxllxkxrxxxpcFNDDiQGTgAVvLaxxxxAxx
ADI76993   .gr..k...d.vqcvkkl..r...vl....glp..rr..dty.prla.....v......t..alss.vr
ODQ72042   .gr..k...d.vqcvkkl..r...vl....glp..rr..dty.prla.....v......t..alss.vr
ADK56109   .gr..k...d.vqcvkkl..r...vl....glp..rr..dty.prla.....v......t..alss.vr
AOW05618   .kq..d.i.n.vqsarrl..k...v.....gla..hki.d.y.pei.............t..sita.lk
XP_504112  .kq..d.i.n.vqsarrl..k...v.....gla..hki.d.y.pei.............t..sita.lk
BBE10583   .ae..k...r.celvree..q...ll....gvs..aki.tty.nkqsv....m...a.....alls.vk
CDR37260   .ae..k...r.celvree..q...ll....gvs..ski.tty.nkqsv....m...a.....alls.vk
PRQ74895   .ae..k...r.celvree..q...ll....gvs..ski.tty.nkqsv....m...a.....alls.vk
KWU45829   .ka..e..ek.cdivred..q...ml....gvs..ar..a.y.piqs.....m...a.....alis.vk
POY70860   .kq..e..ek.cdivred..q...ml....gvs..ar..a.y.piqs.....m...a.....alvs.vk
KPV76802   .ek..q...k.cdivrea..q...ml....avd..sr..q.f.pkqs.....s...a.....alvs.vk
TNY24152   .ek..q...k.cdivrqe..q...ml.l...ssa.smrfvd.y.hkqsi....a...s.....alas.vg
CEQ39123   .ae..a...k.cdlvrem..q...ml....gvs..gr..n.f.pkqs.....m...a....salvs.cr
CED85069   .ki..e.i.k.ikncka1f.d...l.....gls..qr..d.yadei.............t.salma.vg
ANJ02849   eekcle.m.v.mrcmhetf.nm..v.qh..wqtpl.fp..h.n.dly.................gair.fh
PRQ75739   eekcle.m.v.mrcmhetf.nmvqv.qh..wqtpl.fp..h.n.dly.................gair.fh
CDR45495   qekcle.mev.mrcmhetf.nm..v.qh..wqtpl.fp..h.n.dly.................gair.fh
EGU11086   eekcle.m.v.mrcmhetf.nm..v.qh..wqtpl.fp..h.n.dly.................gair.fh
GEM12284   edkcme.mev.mremhktf.nm..v.qh..wqtpl.fp..hrn.dly.................gair.fh
KWU43902   ddkcee...v.mkemhatf.nm..i.q....httl.fp..q.n.dvy.................gair.fh
POY76220   dedcek..ev.mkemhatf.nm..i.q....httl.fp..hnn.eiy...............i..gair.fr
KPV73817   ddkcee.mev.mremhkef.nm..i.q...whttl.fp..h.n.diy.................gair.fa
TNY17208   ddkcee.m.v.mremhkef.nm..i.q...whttl.fp..h.n.diy.................gair.fa
CEQ41698   detcea...v.mkamnkaf.nm..i.q....httl.fp..e.h.ery.................gair.fq
```

Figure S2. MAFFT alignment of malic enzymes showing the divalent ion binding sites [Borsch and Westhoff, 1990]. Identical amino acid residues in all the enzymes are signified by a dot (.) ADI76993, *Lipomyces starkeyi ; ODQ72042, Lipomyces starkeyi NRRL Y-11557; ADK56109, Lipomyces starkeyi; AOW05618, Yarrowia lipolytica CLIB89(W29); XP_504112, Yarrowia lipolytica CLIB122; BBE10583, Rhodotorula toruloides NBRC10032; CDR37260, Rhodotorula toruloides CECT1137; PRQ74895, Rhodotorula toruloides NBRC0880; KWU45829, Rhodotorula sp. JG-1b; POY70860, Rhodotorula taiwanensis; KPV76802, Rhodotorula graminis WP1; TNY24152, Rhodotorula diobovata; CEQ39123, Sporidiobolus salmonicolor; CED85069, Xanthophyllomyces dendrorhous; ANJ02849, Rhodotorula toruloides IFO0880; PRQ75739, Rhodotorula toruloides NBRC0880; CDR45495, Rhodotorula toruloides CECT1137; EGU11086, Rhodotorula toruloides ATCC 204091; GEM12284, Rhodotorula toruloides NBRC10032; KWU43902, Rhodotorula sp. JG-1b; POY76220, Rhodotorula taiwanensis; KPV73817, Rhodotorula graminis WP1; TNY17208, Rhodotorula diobovata; CEQ41698, Sporidiobolus salmonicolor.*

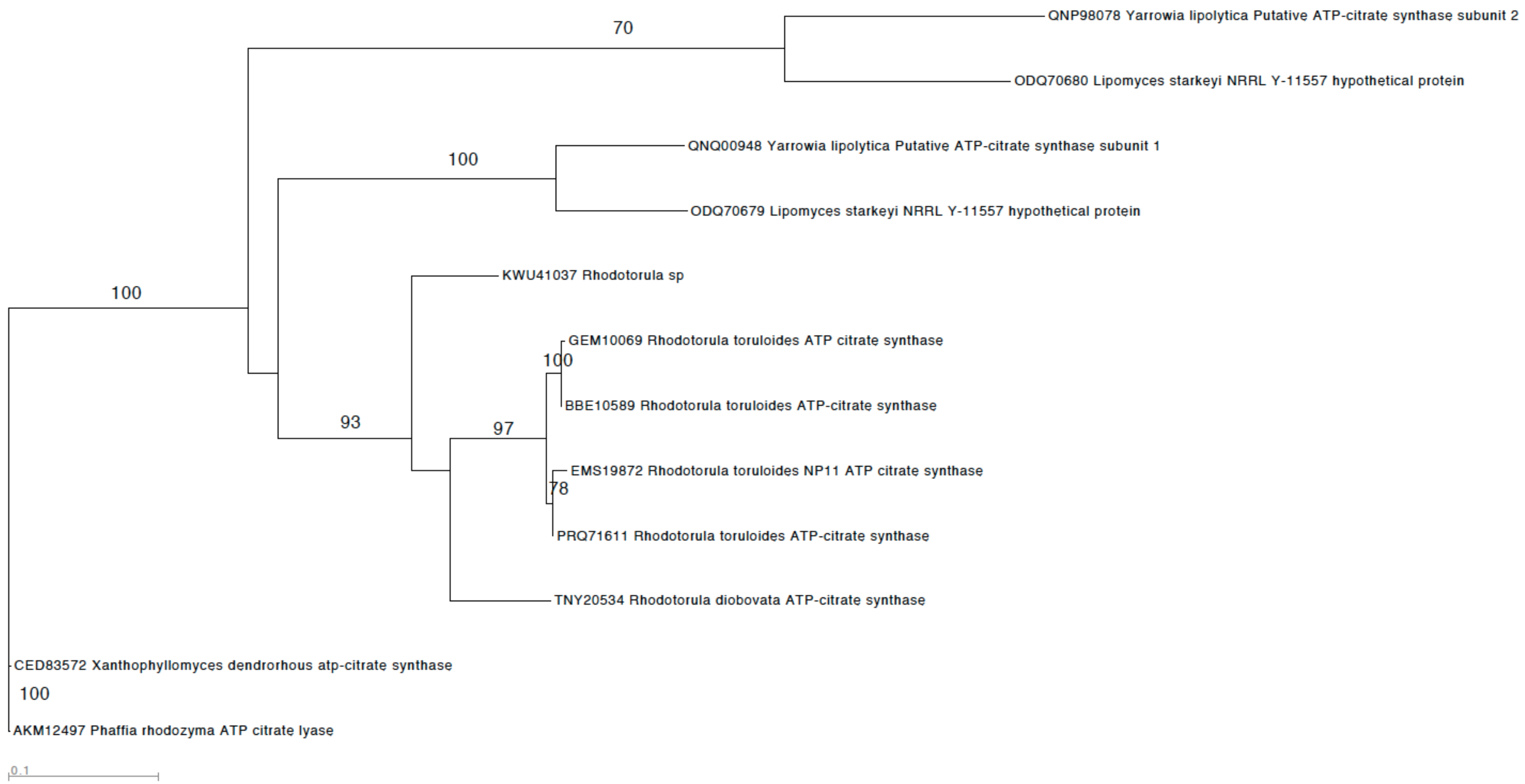

Figure S3. PROML phylogenetic tree constructed from an alignment of ATP:Citrate lyase sequences from *Rhodotorula sp., Yarrowia, Lipomyes,* and *Xanthophyllomyces.* Analysis shows the relationship between the two ACL subunits of *Y. lipolytica* and *L. starkeyi*. The significant nodes on the tree with bootstrap support values > 70% are highlighted.

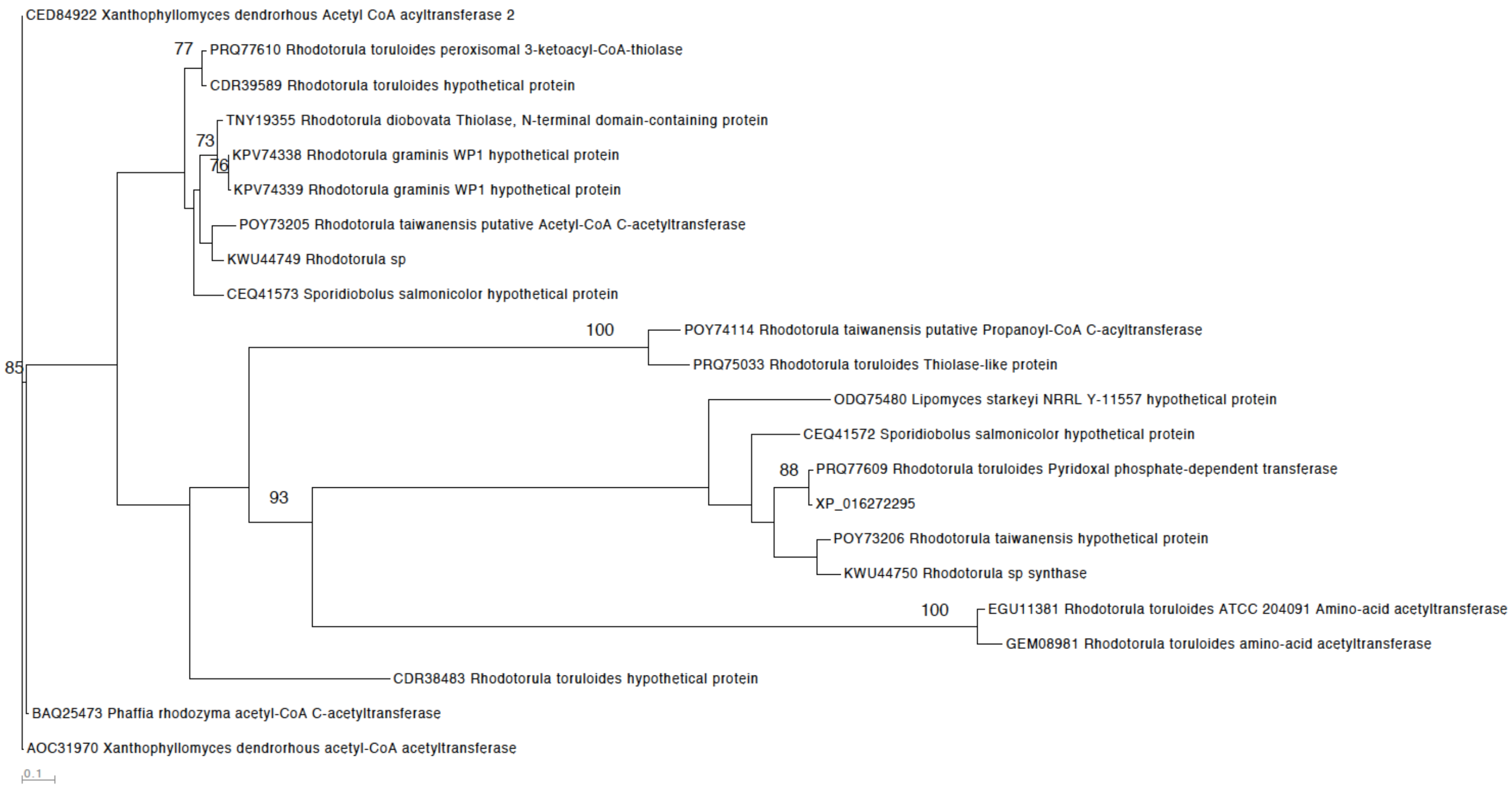

Figure S4. PROML phylogenetic tree constructed from a MAFFT alignment of Acetyl-CoA C-acetyltransferase sequences from *Rhodotorula species, Yarrowia, Lipomyes, Sporidobolus* and *Xanthophyllomyces*. The significant nodes on the tree with bootstrap support values > 70% are highlighted.

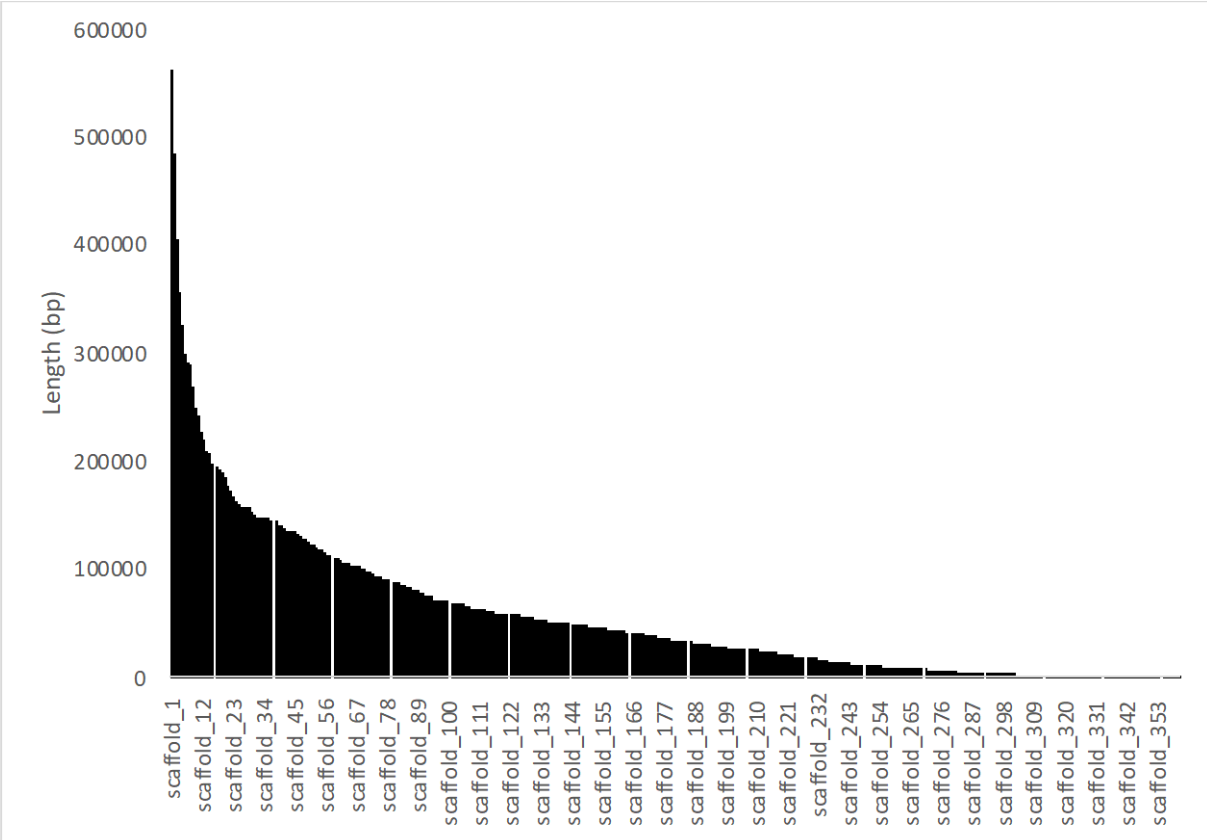

Figure S5. Scaffold length distribution of all 361 scaffolds from the *de novo* assembly of *Rhodotorula diobovata* 08-225.

Table S1. Scaffold length distribution for the 361 scaffolds used to assemble the *Rhodotorula diobovata* genome.

Scaffold	*Length*	*Scaffold*	*Length*	*Scaffold*	*Length*	*Scaffold*	*Length*	*Scaffold*	*Length*	*Scaffold*	*Length*	*Scaffold*	*Length*	*Scaffold*	*Length*
scaffold_1	563716	scaffold_51	124031	scaffold_101	70207	scaffold_151	47520	scaffold_201	27893	scaffold_251	12278	scaffold_301	3813	scaffold_351	1315
scaffold_2	485998	scaffold_52	122682	scaffold_102	69632	scaffold_152	47110	scaffold_202	27855	scaffold_252	12155	scaffold_302	3579	scaffold_352	1288
scaffold_3	407501	scaffold_53	122072	scaffold_103	69347	scaffold_153	46676	scaffold_203	27588	scaffold_253	11161	scaffold_303	3478	scaffold_353	1262
scaffold_4	356559	scaffold_54	120034	scaffold_104	69211	scaffold_154	46507	scaffold_204	27584	scaffold_254	11093	scaffold_304	3335	scaffold_354	1196
scaffold_5	328106	scaffold_55	118648	scaffold_105	68795	scaffold_155	45997	scaffold_205	27231	scaffold_255	10956	scaffold_305	3331	scaffold_355	1182
scaffold_6	300363	scaffold_56	115626	scaffold_106	67249	scaffold_156	45708	scaffold_206	27201	scaffold_256	10836	scaffold_306	3278	scaffold_356	1148
scaffold_7	293503	scaffold_57	114464	scaffold_107	66952	scaffold_157	45575	scaffold_207	27151	scaffold_257	10701	scaffold_307	3271	scaffold_357	1139
scaffold_8	290717	scaffold_58	112899	scaffold_108	65288	scaffold_158	44317	scaffold_208	27066	scaffold_258	10544	scaffold_308	3211	scaffold_358	1128
scaffold_9	271387	scaffold_59	111718	scaffold_109	64973	scaffold_159	44249	scaffold_209	26740	scaffold_259	10276	scaffold_309	3102	scaffold_359	1120
scaffold_10	249850	scaffold_60	110819	scaffold_110	64231	scaffold_160	44153	scaffold_210	25954	scaffold_260	10268	scaffold_310	3078	scaffold_360	1075
scaffold_11	243259	scaffold_61	108476	scaffold_111	64191	scaffold_161	44053	scaffold_211	25607	scaffold_261	10259	scaffold_311	3070	scaffold_361	1004
scaffold_12	226979	scaffold_62	107663	scaffold_112	63824	scaffold_162	43711	scaffold_212	25160	scaffold_262	10071	scaffold_312	3057		
scaffold_13	221070	scaffold_63	106344	scaffold_113	63298	scaffold_163	43639	scaffold_213	25044	scaffold_263	9990	scaffold_313	3033		
scaffold_14	211651	scaffold_64	106129	scaffold_114	62868	scaffold_164	42655	scaffold_214	24880	scaffold_264	9730	scaffold_314	3006		
scaffold_15	206970	scaffold_65	104307	scaffold_115	62290	scaffold_165	42332	scaffold_215	24258	scaffold_265	9721	scaffold_315	2936		
scaffold_16	199045	scaffold_66	103535	scaffold_116	61395	scaffold_166	41672	scaffold_216	24237	scaffold_266	9686	scaffold_316	2918		
scaffold_17	195697	scaffold_67	103456	scaffold_117	60354	scaffold_167	41657	scaffold_217	23663	scaffold_267	9332	scaffold_317	2863		
scaffold_18	193384	scaffold_68	102875	scaffold_118	60277	scaffold_168	41460	scaffold_218	23278	scaffold_268	9308	scaffold_318	2831		
scaffold_19	190360	scaffold_69	102305	scaffold_119	59913	scaffold_169	41325	scaffold_219	23196	scaffold_269	9255	scaffold_319	2815		
scaffold_20	185523	scaffold_70	100411	scaffold_120	59639	scaffold_170	40336	scaffold_220	23024	scaffold_270	8581	scaffold_320	2791		
scaffold_21	178835	scaffold_71	100164	scaffold_121	59502	scaffold_171	40183	scaffold_221	22963	scaffold_271	8234	scaffold_321	2619		
scaffold_22	173499	scaffold_72	98624	scaffold_122	59183	scaffold_172	38870	scaffold_222	22185	scaffold_272	8062	scaffold_322	2561		

scaffold_23	169270	scaffold_73	96503	scaffold_123	59093	scaffold_173	38656	scaffold_223	21937	scaffold_273	7848	scaffold_323	2561
scaffold_24	163365	scaffold_74	95059	scaffold_124	59047	scaffold_174	38429	scaffold_224	19985	scaffold_274	7662	scaffold_324	2541
scaffold_25	161871	scaffold_75	94951	scaffold_125	58310	scaffold_175	38188	scaffold_225	19640	scaffold_275	7603	scaffold_325	2455
scaffold_26	159488	scaffold_76	94947	scaffold_126	57999	scaffold_176	37859	scaffold_226	19609	scaffold_276	7573	scaffold_326	2398
scaffold_27	159370	scaffold_77	92703	scaffold_127	57994	scaffold_177	37811	scaffold_227	19541	scaffold_277	7182	scaffold_327	2354
scaffold_28	159096	scaffold_78	92176	scaffold_128	57347	scaffold_178	37429	scaffold_228	19450	scaffold_278	7022	scaffold_328	2332
scaffold_29	158621	scaffold_79	91617	scaffold_129	56638	scaffold_179	36516	scaffold_229	19432	scaffold_279	6981	scaffold_329	2307
scaffold_30	153256	scaffold_80	88545	scaffold_130	56506	scaffold_180	35512	scaffold_230	18937	scaffold_280	6597	scaffold_330	2306
scaffold_31	150981	scaffold_81	88281	scaffold_131	55444	scaffold_181	35373	scaffold_231	18664	scaffold_281	6112	scaffold_331	2136
scaffold_32	148907	scaffold_82	88211	scaffold_132	55156	scaffold_182	35306	scaffold_232	18241	scaffold_282	6002	scaffold_332	2134
scaffold_33	148760	scaffold_83	87649	scaffold_133	55140	scaffold_183	35020	scaffold_233	17528	scaffold_283	5847	scaffold_333	2110
scaffold_34	148594	scaffold_84	86773	scaffold_134	54795	scaffold_184	34800	scaffold_234	16884	scaffold_284	5792	scaffold_334	2046
scaffold_35	148281	scaffold_85	83878	scaffold_135	54361	scaffold_185	34598	scaffold_235	16141	scaffold_285	5677	scaffold_335	2024
scaffold_36	148251	scaffold_86	83170	scaffold_136	52756	scaffold_186	34189	scaffold_236	15173	scaffold_286	5612	scaffold_336	2016
scaffold_37	145750	scaffold_87	82556	scaffold_137	52428	scaffold_187	32948	scaffold_237	14969	scaffold_287	5555	scaffold_337	1955
scaffold_38	145744	scaffold_88	82150	scaffold_138	52202	scaffold_188	32887	scaffold_238	14778	scaffold_288	5488	scaffold_338	1920
scaffold_39	142015	scaffold_89	82131	scaffold_139	52179	scaffold_189	32159	scaffold_239	14566	scaffold_289	5348	scaffold_339	1893
scaffold_40	140972	scaffold_90	78598	scaffold_140	52053	scaffold_190	32109	scaffold_240	14123	scaffold_290	5080	scaffold_340	1878
scaffold_41	138849	scaffold_91	78428	scaffold_141	52024	scaffold_191	31878	scaffold_241	14118	scaffold_291	4585	scaffold_341	1783
scaffold_42	136445	scaffold_92	76379	scaffold_142	50806	scaffold_192	31807	scaffold_242	13859	scaffold_292	4585	scaffold_342	1733
scaffold_43	135953	scaffold_93	76331	scaffold_143	50753	scaffold_193	31432	scaffold_243	13561	scaffold_293	4539	scaffold_343	1644
scaffold_44	135147	scaffold_94	75530	scaffold_144	50418	scaffold_194	30795	scaffold_244	13431	scaffold_294	4397	scaffold_344	1615
scaffold_45	135023	scaffold_95	72949	scaffold_145	50179	scaffold_195	30199	scaffold_245	13142	scaffold_295	4396	scaffold_345	1560
scaffold_46	133771	scaffold_96	71833	scaffold_146	50109	scaffold_196	29853	scaffold_246	13101	scaffold_296	4353	scaffold_346	1541

scaffold_47	130143	scaffold_97	71784	scaffold_147	49962	scaffold_197	29742	scaffold_247	13100	scaffold_297	4278	scaffold_347	1538
scaffold_48	128697	scaffold_98	71720	scaffold_148	49783	scaffold_198	29437	scaffold_248	12984	scaffold_298	4261	scaffold_348	1494
scaffold_49	128532	scaffold_99	71082	scaffold_149	49600	scaffold_199	28908	scaffold_249	12429	scaffold_299	4090	scaffold_349	1457
scaffold_50	125606	scaffold_100	71010	scaffold_150	48167	scaffold_200	27926	scaffold_250	12336	scaffold_300	3987	scaffold_350	1397

Table S2. Genome coverage calculation for Illumina and Ion Torrent reads.

GENOME COVERAGE

Desired coverage (50-fold)	Illumina	IT
genome size G	21000000	21000000
avg. read size	144	305
probability of coverage P	0.99	0.99
fraction of genome per read f	6.86E-06	1.45E-05
desired coverage C	50	50
1-fold coverage ln(1-P)/ln(1-f)	671585.016	317074.9889
req. reads N = C[ln(1-P)/ln(1-f)]	33579250.8	15853749.45

Actual coverage

Illumina

file	format	type	num_seqs	sum_len	min_len	avg_len	max_len
DL300_S1_L001_R1_001.fastq.gz	FASTQ	DNA	4878013	713623894	30	146.3	151
DL300_S1_L001_R2_001.fastq.gz	FASTQ	DNA	4878013	703035995	30	144.1	151
DL400_S2_L001_R1_001.fastq.gz	FASTQ	DNA	4003821	580031498	30	144.9	151
DL400_S2_L001_R2_001.fastq.gz	FASTQ	DNA	4003821	568995556	30	142.1	151
DL700_S3_L001_R1_001.fastq.gz	FASTQ	DNA	2830249	412524162	30	145.8	151
DL700_S3_L001_R2_001.fastq.gz	FASTQ	DNA	2830249	401509203	30	141.9	151
total			23424166	3379720308			
Illumina coverage = total seqs/1-fold C			34.87892885				

Ion Torrent

file	format	type	num_seqs	sum_len	min_len	avg_len	max_len
Pollux-corrected IT reads	FASTQ	DNA	2965701	904538805	25	305	529
IT coverage = total seqs/1-fold C			9.353311058				
Total Illumina + IT coverage			44.23223991				
